# A 5-nV/√Hz Chopper Negative-R Stabilization Preamplifier for Audio Applications

**DOI:** 10.3390/mi11050478

**Published:** 2020-05-02

**Authors:** Jamel Nebhen, Khaled Alnowaiser, Stephane Meillere

**Affiliations:** 1 College of Computer Engineering and Sciences, Prince Sattam bin Abdulaziz University, P.O. Box 151, Alkharj 11942, Saudi Arabia; k.alnowaiser@psau.edu.sa; 2 Aix Marseille University, CNRS, IM2NP UMR 7334, 13451 Marseille, France; s.meillere@im2np.fr

**Keywords:** preamplifier, chopper stabilization, negative-R, CMOS operational transconductance amplifier (OTA), 1/f noise

## Abstract

This paper presents a low-noise and low-power audio preamplifier. The proposed low-noise preamplifier employs a delay-time chopper stabilization (CHS) technique and a negative-R circuit, both in the auxiliary amplifier to cancel the non-idealities of the main amplifier. The proposed technique makes it possible to mitigate the preamplifier 1/*f* noise and thermal noise and improve its linearity. The low-noise preamplifier is implemented in 65 nm complementary metal-oxide semiconductor (CMOS) technology. The supply voltage is 1.2 V, while the power consumption is 159 µW, and the core area is 192 µm^2^. The proposed circuit of the preamplifier was fabricated and measured. From the measurement results over a signal bandwidth of 20 kHz, it achieves a signal-to-noise ratio (SNR) of 80 dB, an equivalent-input referred noise of 5 nV/√Hz and a noise efficiency factor (NEF) of 1.9 within the frequency range from 1 Hz to 20 kHz.

## 1. Introduction

The Internet of Things (IoT) is now recognized by the industry, and in particular the electronics industry, as one of the main engines of growth for the decade to come, if not longer. The IoT refers to any application taking advantage of the networking of objects capable of interacting with their environment to measure key parameters of this environment, transmitting this data for analysis, sometimes in real time, and making decisions to control or optimize a system. Detection is the starting point for the IoT and smart home applications. It is also the first problem faced by maker followers and professional designers. The design of many economical transducers such as accelerometers, force sensors, extensometers and pressure transducers is based on resistive Wheatstone bridges for differential voltages in millivolts (mV). Before going into detail, it is essential to accurately capture these low-level signals and amplify them to levels compatible with analog-to-digital converters (ADCs), without DC offset or noise. Likewise, current detection using high potential ammeter shunts requires amplifiers without inputs referenced to ground, and capable of tolerating high common mode voltages. Micro-Electro-Mechanical Systems (MEMS) are sensors or actuators whose lateral dimensions and thickness are of the order of a micrometer. For decades, and still today, MEMS sensors have been manufactured on a large scale for many consumer applications, such as aerospace [1]; inertial sensors in mobile phones, such as gyrometers and accelerometers [2,3]; video game controllers; and airbag triggers. These devices, which are the basis of research tools [4], have reached a sufficient maturity to be directly developed and integrated by large industrial groups, such as STMicroelectronics [5].

In the world of transistors, it is known that the reduction of dimensions mainly makes it possible to integrate more devices on a given surface. Therefore, it enables a reduction of the manufacturing cost of the transistor, to increase the performance of the integrated circuit and to reduce the operating voltage. Regarding sensors, reduced dimensions are other benefits that enabled the development of emerging applications. Therefore, since the 2000s, these sensors are reduced to the nanometer scale with the name “Nano-Electro-Mechanical Systems” (NEMS). These devices allow the study and detection of objects at the molecular scale [6,7] and also at the quantum scale [8]. The constant times are likewise reduced, which implies a limited response time, only because of the electronics control and not the NEMS itself. In addition, in the nanoscale era, we can see a modification in the intrinsic properties of materials as in silicon nanowires, and their thermal and conductive properties modified by the size effect. Hearing implants are technological devices developed to correct hearing loss. Today, the cochlear implant is the most complete system. It implements the fields of acoustics, electronics, signal processing and information, biology, and knowledge of the human physiology. The objective of the microphone is to transduce acoustic waves to an electrical signal. Consequently, it operates in the frequency range from 20 Hz to 20 kHz [9]. The low noise amplifier (LNA) is one of the key devices of an audio system. Located just after the audio sensor, the LNA receives a variety of low-level signals from this sensor. The LNA is placed on top of the audio conditioning chain. Therefore, it must reach a very important signal-to-noise ratio (SNR) output, to facilitate the processing of the audio signal information by the downstream components [10]. The major function of the LNA consists of amplifying the useful signal, while minimizing the noise contribution, and thus allowing the detection of signals at very low powers. Therefore, its linearity, selectivity, lower consumption and reduced surface require a very fine design [11].

There are three types of CMOS dynamic offset cancellation techniques: trimming, chopping, and auto-zeroing [12,13,14]. To eliminate offset during production, trimming technique is usually performed. Chopping involves modulating offset and 1/*f* noise to a chopping frequency, leaving only white noise in the base band. However, due to the up-modulated offset and 1/*f* noise, a chopper ripple appears at the amplifier’s output. Auto-zeroing involves sampling the offset and low frequency 1/*f* noise on an auto-zero capacitor, and then subtracted in subsequent clock phases. However, the noise folding associated with sampling results in increased base band noise [15]. Thus, to achieve high power efficiency, chopping is the technique of choice, provided that associated ripple can be sufficiently suppressed. Chopper amplifiers are widely adopted in these systems for their advantageous low noise, high input impedance, and high common mode rejection ratio (CMRR). Various types of amplifiers for bio-potential measurements have been reported [16,17,18,19,20,21,22,23]. The amplifier reported in [17,18] used a capacitive feedback network and chopper modulation method with a DC servo loop for 1/*f* noise reduction, but it required a very large capacitor array, which significantly increased its die area [17]. The amplifier reported in [19,20] employed a chopper implemented inside of the feedback loop and thus suffered from CMRR reduction. The instrumentation amplifier reported in [21,22] overcame the CMRR reduction problem with an input impedance boosting loop that added the expense of extra circuitry.

In this paper, we present a high-linear chopper negative-R stabilization audio preamplifier, which employs two proposed techniques to reduce the 1/*f* noise and the thermal noise at the same time. The two proposed techniques are the delay-time chopper stabilization (CHS) technique [24,25] and the negative-R technique [26]. The paper is organized as follows. Section 2 analyses the delay-time chopper stabilization circuit. Section 3 analyses the chopper negative-R circuit. Section 4 describes the CMOS implementation of the LNA. Section 5 presents the simulation results. Finally, the conclusion is drawn in Section 6.

## 2. Analysis of the Delay-Time Chopper Stabilization Preamplifier

**Figure 1:**
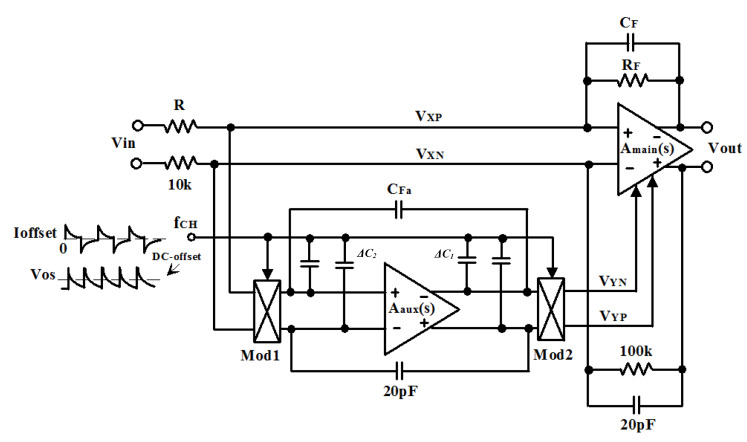
Chopper stabilization preamplifier.

To reduce the input-equivalent noise of the preamplifier, we propose to use the well-known CHS technique. The CHS preamplifier circuit is shown in Figure 1 [27]. It is composed by the main amplifier, *A*_main_(*s*), and by the auxiliary amplifier, *A*_aux_(*s*). The non-idealities of *A*_main_(*s*) in low-frequency path are canceled by the *A*_aux_(*s*). Firstly, the input signal at *A*_main_(*s*) is measured by *A*_aux_(*s*). Then, the virtual ground *V_X_ = V_XP_ − V_XN_* is derived toward zero by *A*_aux_(*s*). Therefore, a compensation voltage *V_Y_ = V_YP_ − V_YN_* is generated by the *A*_aux_(*s*) to the *A*_main_(*s*). The virtual ground *V_X_* can be written as

(1) $$V_{X}=\frac{V_{\mathrm{OUT}}}{\left[A_{\mathrm{main}}(s)\cdot \left(1+A_{\mathrm{aux}}(s)\right)\right]},$$

Namely, the auxiliary amplifier allows attenuating the main amplifier noise portion as

(2) $$v_{n,\mathrm{main}\text{\_}\mathrm{in}}^{2}=\frac{v_{n,\mathrm{main}0\text{\_}\mathrm{in}}^{2}}{{\left|A_{\mathrm{aux}\text{\_}\mathrm{int}}(s)\right|}^{2}},$$

where $v_{n,\mathrm{main}\_\mathrm{in}}^{2}$ is the input noise of the main amplifier with the auxiliary amplifier and $v_{n,\mathrm{main}0\_\mathrm{in}}^{2}$ is the input noise of the main amplifier without the auxiliary amplifier. *A*_aux_int_(*s*) represents the active RC-integrator signal transfer function (STF), which can be written as

(3) $$A_{\mathrm{aux}\text{\_}\mathrm{int}}(s)=\frac{A_{\mathrm{aux}}(s)}{\left[1+sRC_{Fa}\cdot \left(1+A_{aux}(s)\right)\right]}.$$

The signal path mismatch and the demodulated current spikes generate a residual offset *V*_os_. Therefore, AC current spike is caused by the mismatch between the capacitances, due to clock feed-through at the chopper clocks transition moments. The first modulator Mod1 rectify this AC current. Therefore, a DC spike current appears at its input. The resulting DC spike current has an average value *I*_offset_ of

(4) $$I_{\mathrm{offset}}=2\left(\Delta C_{1}-\Delta C_{2}\right)V_{\mathrm{clk}}f_{\mathrm{CH}},$$

where Δ*C*_1_ and Δ*C*_2_ denote the CHS mismatch parasitic capacitance, *V*_clk_ denotes the clock signal magnitude, and *f*_CH_ denotes the Chopping clock frequency. The chopper series impedance and the input signal source are going through by this noise current. Therefore, it depicts as an input voltage spike. The residual offset *V*_os_ resulting from the spike average DC value can be written as

(5) $$V_{\mathrm{os}}=2R\left(\Delta C_{1}-\Delta C_{2}\right)V_{\mathrm{clk}}f_{\mathrm{CH}},$$

where *R* denotes the equivalent input impedance. Therefore, a residual offset *V*_os_ depicts the spike average DC value. Moreover, a spike voltage *V*_os_ is created in the input of the Mod1. This spike voltage causes a low-frequency interference.

**Figure 2:**
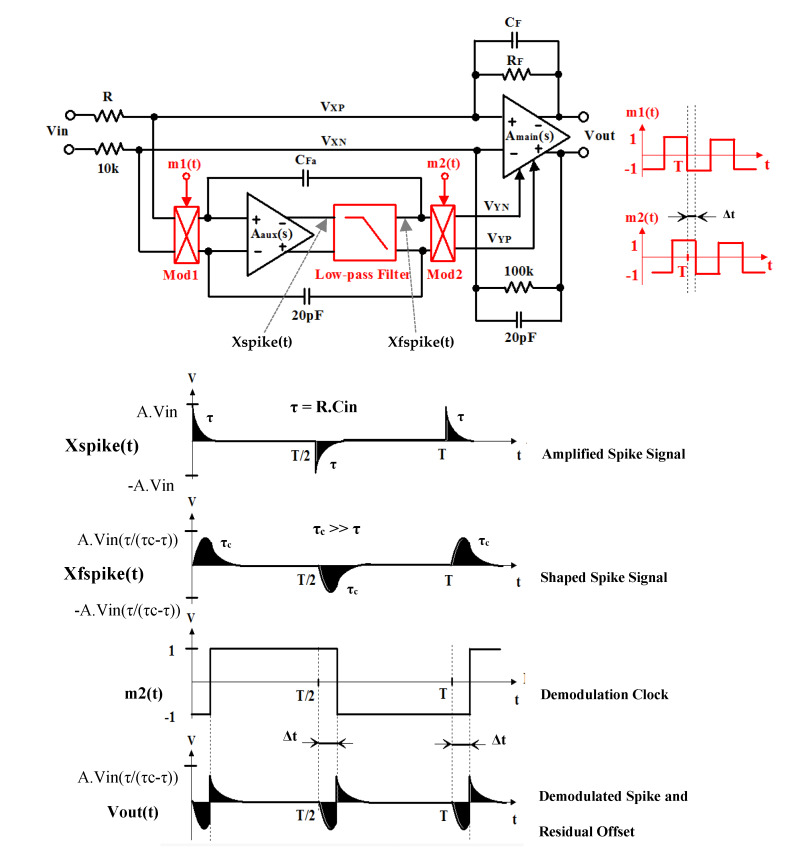
Delay-time chopper stabilization preamplifier.

To cancel out this interference, we propose to create a proper delay Δ*t* between Mod1 and Mod2. The proposed preamplifier CHS technique is shown in Figure 2. The auxiliary amplifier is located between two modulating clock signals: m1(t) and m2(t) with period *T*. Moreover, we introduce a delay ∆*t* between the two clock signals m1(t) and m2(t) at the same time. Due to the introduction of the delay ∆*t*, this technique causes a chopping of the spike signal itself. Therefore, the DC content of the output signal *V*_out_(t) is minimized. The residual output DC offset is completely cancelled if an optimum delay value ∆*t*_opt_ exists, which can be written as

(6) $$\Delta t_{\mathrm{opt}}=\ln\left(2\right)\times \tau ,$$

where *τ = R ×* C_in_ with *R*, and C_in_ denotes respectively the input resistance and the amplifier’s input capacitance.

The major weakness of this technique is the *τ* itself, which not only depends on the amplifier’s source resistance *R*, but also on its input capacitance C_in_. The input preamplifier’s spike signal is amplified and then multiplied with m2(t). The resulting output signal *V_Y_* then contains, apart from higher order harmonics of the chopping frequency, a DC part or residual offset, which is due to chopping artifacts. To solve this problem, shaping of the spike can be introduced by the addition of a first order low-pass filter with time constant *τ_c_* after *A*_aux_(*s*). We must have *T >> τ_c_ >> τ* with *T* is the period of the square wave signal m1(t). The shape of the time response of the filtered spike is primarily determined by *τ_c_*. Since the output offset is still linearly dependent on *τ*, the optimization of ∆*t*_opt_ has been done in such a way that offset reduction is most effective for a worst-case preamplifier resistance. For our specific implementation ∆*t*_opt_*/τ_c_* = 0.8 has been chosen. The low-pass filter has a cut-off frequency of 300 kHz. The nominal chopping frequency *f*_CH_ is 120 kHz.

## 3. Analysis of the Chopper Negative-R Stabilization Preamplifier

**Figure 3:**
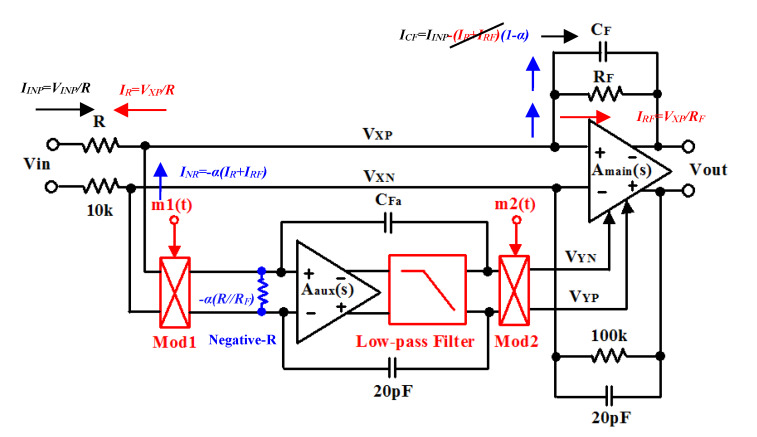
Chopper negative-R stabilization preamplifier.

The delay-time chopper stabilization removes only the 1/*f* noise of the preamplifier. However, the chopper does not affect the *A*_aux_(*s*) thermal noise, and the overall noise level of the preamplifier is not reduced. Likewise, a large bandwidth of *A*_aux_(*s*) is required in order to keep its high gain at *f*_CH_. To reduce both the 1/*f* noise and the thermal noise, we propose to use the chopper negative-R stabilization circuit as shown in Figure 3. This proposed technique allows removing the preamplifier non-ideality at *V_X_*. It will compensate for the error current I_R_ and I_RF_ generated by *R* and *R*_F_, respectively at *V_X_*. Therefore, we choose the negative-R value in order to matching the value of the parallel resistors *R*//*R*_F_. As a result, the chopper negative-R stabilization technique allows mitigating the *V_X_* error. In the first case, the preamplifier noise is analyzed without the negative-R circuit. The overall main and auxiliary opamps noise transfer function (NTF) can be written as

(7) $$\frac{v_{n,\mathrm{in}}^{2}}{v_{n,\mathrm{opamps}}^{2}}={\left(1+\frac{R}{R_{F}}\right)}^{2}{\left[1+s\left(R∥R_{F}\right)C_{F}\right]}^{2},$$

where $v_{n,\mathrm{in}}^{2}$ denotes the input-referred noise of preamplifier, and $v_{n,\mathrm{opamps}}^{2}$ denotes the noise of opamps. The $v_{n,\mathrm{opamps}}^{2}$ noise are calculated at the preamplifier input node. In the second case, the preamplifier noise is analyzed with the chopper negative-R circuit. The overall main and auxiliary opamps NTF becomes

(8) $$\frac{v_{n,\mathrm{in}}^{2}}{v_{n,\mathrm{opamps}}^{2}}={\left(1+\frac{R}{R_{F}}\right)}^{2}{\left[1+s\left(R∥R_{F}\right)C_{F}\right]}^{2}{\left(\frac{\alpha -1}{\alpha }\right)}^{2},$$

where the very important coefficient *α* denotes the negative-R matching coefficient of the parallel resistors *R*//*R*_F_. If *α* becomes closer to 2, ideal compensation for *α =* 1, the noise opamps decreases.

However, the negative-R circuit adds an amount of noise of $2\cdot \left(R/R_{N}\right)\cdot 4{\mathrm{kTR}}_{N}$, with $R_{N}=-\propto \left(R∥R_{F}\right)$ in this case. As a result, the proposed negative-R preamplifier has an input-referred noise of

(9) $$v_{n,\mathrm{in}}^{2}=2\times \left[4kTR+4kTR_{F}{\left(\frac{R}{R_{F}}\right)}^{2}\right]+{\left|H(s)\right|}^{2}\times v_{n,\mathrm{opamps}}^{2},$$

where *|H(s)|*^2^ denotes the NTF of the negative-R opamps of Equation (8). Therefore, the chopper negative-R circuit allows attenuating the opamps mismatch and offset.

## 4. CMOS Amplifier Implementation

**Figure 4:**
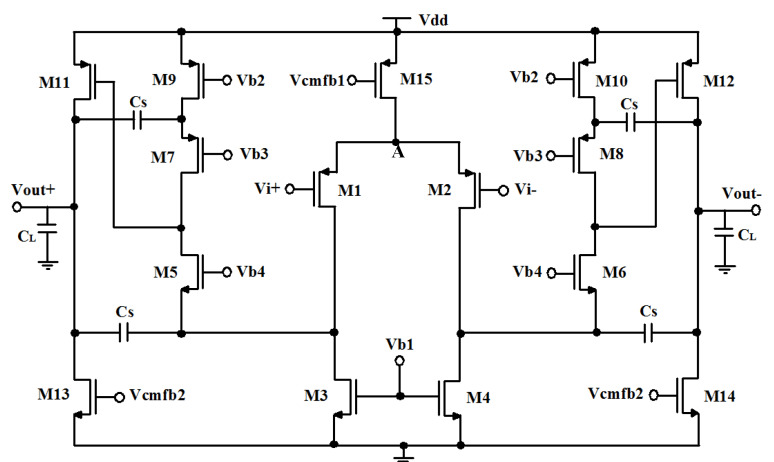
Folded-cascode operational transconductance amplifier (OTA) circuit.

If the amplifier is designed with a single-stage topology under low-voltage operation, then its output swing and its gain are limited. Usually, an amplifier with a two-stage topology, as shown in Figure 4, is used to increase the gain and the output swing. The first stage of the amplifier contributes to the total gain while the second stage contributes to enable a large output swing. The compensation capacitor (*C_S_*) allows stabilizing the two-stage amplifier. In order to improve the frequency response, a resistor is connected in series with the capacitor *C_S_*. Therefore, the amplifier receives a left half plane zero [28].

A desired gain-bandwidth (GBW) and a load capacitance (*C_L_*) are considered to evaluate the amplifier power consumption. If all amplifier transistors have the same overdrive voltage $V_{\mathrm{OV}}=V_{\mathrm{GS}}-V_{T}$, and if the output non-dominant pole is at least three times of the GBW, then the current consumption *I*_two-stage_ of the two-stage operational transconductance amplifier (OTA) can be written as [29,30].

(10) $$I_{\mathrm{two}-\mathrm{stage}}=2\pi \times GBW\times V_{\mathrm{OV}}\times \left(4C_{S}+3C_{L}\right).$$

From Equation (10), it is clear that the maximum of the current is used to drive the compensation capacitors.

Figure 4 shows the schematic of two-stage amplifier. The first stage is a differential OTA providing high gain, while the second stage is configured as a simple common-source stage to give maximum output swing. In contrast to cascode opamps, this topology isolates the gain and output swing requirements [29,30]. The DC-gain Ad and GBW expressions of the amplifier is given as

(11) $$A_{d}=g_{m1}\left(r_{o1}\times r_{o2}\right)\times g_{m3}\left(r_{o3}\times r_{o11}\right),$$

(12) $$GBW=\frac{g_{m1}g_{m3}\left(r_{o1}\times r_{o2}\right)}{2\pi \times C_{L}},$$

where *g_m_*_1_ and *g_m_*_3_ denote the transconductance of transistor M1 and M3, *r_0_*_1_, *r_0_*_2_, *r_0_*_3_, and *r_0_*_11_ denote the output resistance of transistors M1, M2, M3, and M11, respectively. Assuming all the transistors have the same overdrive voltage, the transconductance *g_m_* of M1 and M3 can be written as

(13) $$g_{m1}=\frac{2I_{D1}}{V_{\mathrm{GS}}-V_{T}},$$

(14) $$g_{m3}=\frac{2I_{D3}}{V_{\mathrm{GS}}-V_{T}},$$

where *I*_D_ denotes the drain current, *V*_T_ denotes the threshold voltage, and *V*_GS_ denotes the gate-source voltage of the MOSFET. Further, assuming all branches have the same current and ignoring parasitic capacitance at node *A*, Equation (12) can be modified as

(15) $$GBW=\frac{g_{m}^{2}\left(r_{o1}\times r_{o2}\right)}{2\pi \times C_{L}}=\frac{g_{m}^{2}}{2\pi \times C_{L}\left(\lambda_{n}+\lambda_{p}\right)\times I_{D}},$$

where *λ* denotes the channel-length modulation parameter of the MOSFET. Combining Equations (13)–(15), the total current of the two-stage OTA is then

(16) $$I_{\mathrm{two}-\mathrm{stage}}=GBW\times \pi \times {\left(V_{\mathrm{GS}}-V_{T}\right)}^{2}\times \left(\lambda_{n}+\lambda_{p}\right)\times \left(2C_{L}\right),$$

Since the parasitic capacitor at node *A* is negligible, for more power saving, the frequency compensation is performed by the load capacitance, and no miller compensation is used. The maximum output swing that this amplifier can achieve is $V_{\mathrm{DD}}-\left|V_{\mathrm{overdrive}4}\right|-V_{\mathrm{overdrive}5}$. To achieve a higher signal swing, the overdrive voltage of transistors M4 and M5 are minimized in this design. The two-stage OTA has an open-loop gain of 65-dB, a phase margin of 62°, and a GBW of 16-MHz, with a power consumption of only 72-μW.

The noise sources of the CMOS operational amplifier originate from flicker noise and thermal noise components. The flicker noise component is usually larger than the thermal noise component for frequencies from 1 Hz to 20 kHz for a typical bias conditions and device geometries. The total noise current of a MOSFET is given as [30]

(17) $$\frac{\bar{i_{t}^{2}}}{\Delta f}=\frac{K_{F}g_{m}^{2}}{C_{OX}WLf}+\frac{8KTg_{m}}{3}.$$

Neglecting the thermal noise contribution, the total noise current can be approximated as:

(18) $$\frac{\bar{i_{t}^{2}}}{\Delta f}=\frac{K_{F}g_{m}^{2}}{C_{\mathrm{OX}}WLf},$$

with:

(19) $$g_{m}=\sqrt{2µC_{OX}\frac{W}{L}I_{D}},$$

where *K*_F_ is the flicker noise coefficient, *g_m_* is the transconductance parameter of the MOSFET device, *C_OX_* is the gate-oxide capacitance per unit area, *W* is the channel width, *L* is the channel length, *f* is the frequency, *m* represents the effective mobility, *I*_D_ is the drain current, and Δ*f* is the bandwidth. Thus, the equivalent input-referred voltage noise can be written as:

(20) $$\frac{\bar{v_{eq}^{2}}}{\Delta f}\approx \frac{K_{F}}{C_{\mathrm{OX}}WLf}.$$

Noise analysis of the two-stage operational amplifier of Figure 4 yields

(21) $$\frac{\bar{v_{eq}^{2}}}{\Delta f}\approx 2\left[\bar{v_{n1}^{2}}+\bar{v_{n3}^{2}}{\left(\frac{g_{m3}}{g_{m1}}\right)}^{2}\right].$$

where $\bar{v_{n1}^{2}}$ denotes the thermal noise of transistor M1, and $\bar{v_{n3}^{2}}$ denotes the thermal noise of transistor M3. Substituting Equations (19) and (20) into Equation (21), we have

(22) $$\frac{\bar{v_{eq2S}^{2}}}{\Delta f}\approx \frac{2K_{F}}{C_{\mathrm{OX}}W_{1}f}\left[\frac{1}{L_{1}}+\left(\frac{K_{F3\mu n}}{K_{F1\mu p}}\right)\cdot \frac{L_{1}}{L_{3}^{2}}\right],$$

where the symbols have their usual meanings, and the subscript n represents n-channel device, and subscript p represents p-channel device. It is apparent from Equation (22) that there exists a minimum as *L*_1_ varies. For very low values of *L*_1_, the first term is dominant, whereas for large *L*_1_ values, the second term is dominant. Although the low-noise design practice adapts long channel length *L*_3_ for active load and short channel length *L*_1_ for good phase margin.

**Figure 5:**
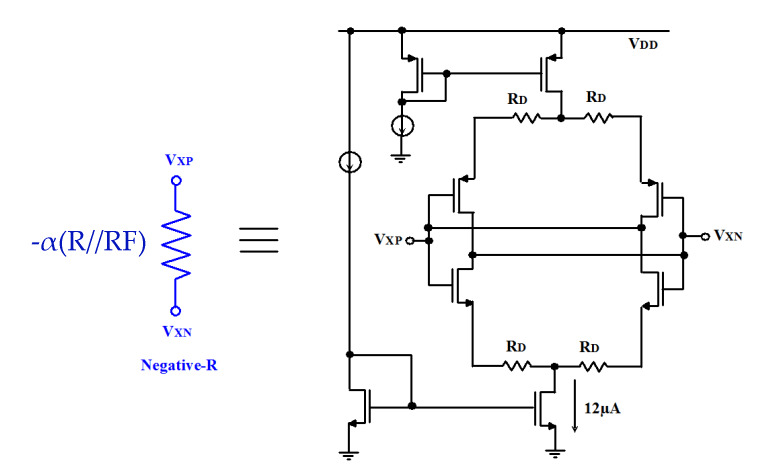
Negative-R circuit.

The transistor-level circuit of the negative-R is shown in Figure 5. It is implemented in a topology of MOSFET source-degeneration. It consumes only 12-μW. In this topology, the degeneration resistor and the current source allows enhancing the accuracy of matching the circuit within 10%. Moreover, a stable *g_m_* of the negative-R is maintained over the temperature and power supply variations [26].

## 5. Measurement Results

**Figure 6:**
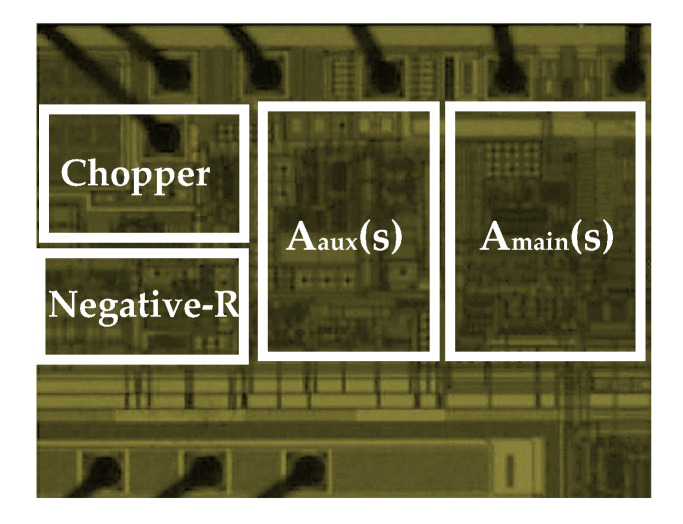
Chip microphotograph of the proposed chopper negative-R stabilization preamplifier in 65 nm CMOS process with an area of 192 µm^2^.

**Figure 7:**
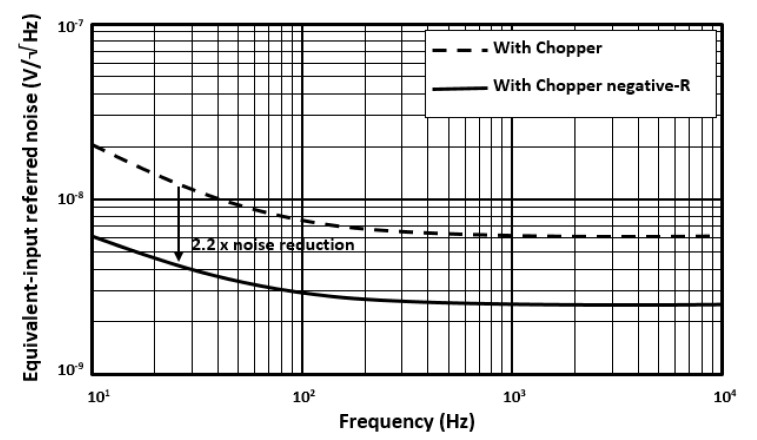
Equivalent-input referred noise of the proposed chopper negative-R stabilization preamplifier.

**Figure 8:**
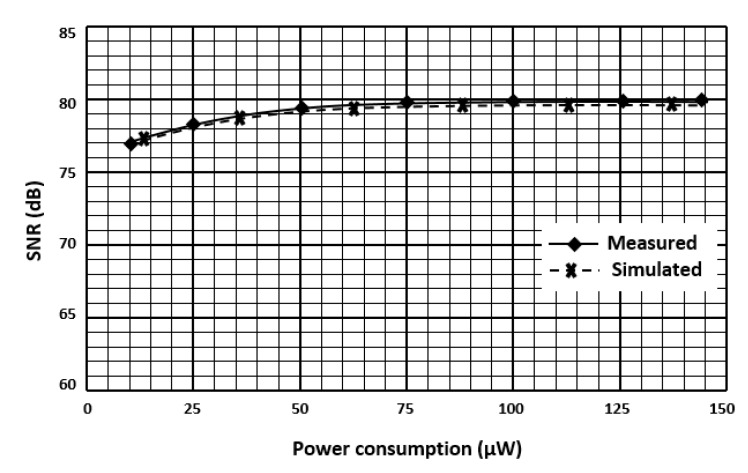
Signal-to-noise ratio (SNR) of the proposed chopper negative-R stabilization preamplifier versus power consumption.

Prototypes of the low-noise preamplifier are fabricated and experimentally characterized. The die microphotograph of the preamplifier is shown in Figure 6. The measurement result of the preamplifier input-referred noise with CHS circuit and with chopper negative-R circuit is shown in Figure 7. The preamplifier has a bandwidth of 20 kHz. From measurement result, if the preamplifier is with CHS circuit, then its input-referred noise is 11 nV/√Hz. If the preamplifier is with chopper negative-R circuit, then its input-referred noise is almost limited by the R, R_F_, and negative-R thermal noise and it is attenuated to 5 nV/√Hz. Moreover, it is clear from measurement result that the chopper negative-R circuit allows to mitigate the opamps 1/*f* noise and thermal noise. As a result, the proposed chopper negative-R stabilization preamplifier allows a noise reduction of 2.2 times, compared to the conventional preamplifier. The curves in Figure 8 show the SNR of the preamplifier as a function of the power consumption. Simulation results have been added for comparison. It appears that the evolution of the experimentally measured SNR is consistent with that obtained with the simulation results. The maximum measured SNR is 80 dB. This value is nearly the same as the simulation result. It is obtained for a current of 132 μA. It corresponds to a power consumption of 159 µW.

The measurement results of the proposed low-noise preamplifier and the comparison with other amplifiers is reported in Table 1. A fundamental parameter is used to evaluate the overall power efficiency of the preamplifier. This parameter is the noise efficiency factor parameter (NEF), which can be written as [31]

(23) $$NEF=V_{\mathrm{rms}}\sqrt{\frac{2\times I_{\mathrm{total}}}{\pi \times U_{\mathrm{th}}\times 4\mathrm{kT}\times BW}},$$

where *I*_total_ is the total current consumption, *V*_rms_ is the root-mean square (RMS) input-referred noise, *U*_th_ is the thermal voltage, and *BW* is the bandwidth of the preamplifier. From Equation (23), it is clear that the NEF parameter includes almost every performance shown in Table 1, namely the equivalent-input referred noise, the power consumption, the bandwidth and indirectly the power-supply rejection ratio (PSRR), and the common-mode rejection ratio (CMRR). In [32,33], the preamplifier has a high CMRR and high PSRR. However, it has also a high equivalent-input referred noise at list of about 18 nV/√Hz. In [22,23], the measured preamplifier has a low CMRR and low PSRR. Moreover, it has a worse equivalent-input referred noise at least 47 nV/√Hz. Therefore, this noise level affects drastically the preamplifier and degrades its performances. As a result, all compared preamplifiers have an equivalent-input referred noise greater than 18 nV/√Hz. On the other hand, our measured preamplifier has a high CMRR and high PSRR. Moreover, it has the lowest equivalent-input referred noise of only 5 nV/√vHz. As a result, for the same performances, our preamplifier has a good tradeoff between the supply voltage, the PSRR, and the CMRR. Our proposed circuit achieves a NEF of 1.9, a PSRR of 110 dB and a CMRR of 121 dB. Therefore, it proves a competitive performance compared to the state-of-the art.

## 6. Conclusions

Two proposed techniques to reduce 1/*f* noise, thermal noise and power consumption of an audio preamplifier are presented in this paper. The preamplifier includes a chopper negative-R stabilization preamplifier, which employs a delay-time chopper stabilization and a negative-R circuit both in the auxiliary amplifier path to cancel the non-idealities of the main amplifier. The low-noise preamplifier is implemented in a 65 nm CMOS technology. The supply voltage is 1.2 V while the power consumption is 159 µW and the core area is 192 µm^2^. The low-noise preamplifier was fabricated and measured. From measurement results over a signal bandwidth of 20 kHz, the proposed preamplifier achieves an SNR of 80 dB, and an equivalent input-referred noise of 5 nV/√Hz. The proposed chopper negative-R stabilization technique mitigates the requirements including thermal noise, 1/*f* noise, and linearity, thus reducing the power dissipation of the preamplifier.

## Figures and Tables

**Table 1:** Performances Comparison of the Proposed Preamplifier with the State-of-the Art.

Specifications	[32]	[33]	[23]	[22]	This Work
Technology, nm	180	320	130	40	65
Supply, V	3.3	3.3	1.2	1.2	1.2
Power, µW	558	561	3	2	159
CMRR, dB	162	120	85	87	121
PSRR, dB	111	115	-	-	110
Bandwidth, kHz	59	40	5	20	20
Technique of noise reduction	Chopper	Chopper	Chopper	Chopper	Chopper-negative R
Noise, nV/√Hz	28.3	18	47	110	5
NEF	4.2	10.6	3.9	4.9	1.9
